# Cafeteria Diet and High-Fructose Rodent Models of NAFLD Differ in the Metabolism of Important PUFA and Palmitoleic Acid without Additional Influence of Sex

**DOI:** 10.3390/nu12113339

**Published:** 2020-10-30

**Authors:** Tomislav Mašek, Josip Barišić, Vedran Micek, Kristina Starčević

**Affiliations:** 1 Department of Animal Nutrition and Dietetics, Faculty of Veterinary Medicine, University of Zagreb, Heinzelova 55, 10000 Zagreb, Croatia; 2 Laboratory for Biotechnology in Aquaculture, Division of Materials Chemistry, Ruđer Bošković Institute, Bijenička cesta 54, 10000 Zagreb, Croatia; josip.barisic@irb.hr; 3 Institute for Medical Research and Occupational Health, Ksaverska cesta 2, 10000 Zagreb, Croatia; vmicek@imi.hr; 4 Department of Chemistry and Biochemistry, Faculty of Veterinary Medicine, University of Zagreb, Heinzelova 55, 10000 Zagreb, Croatia; kristina.starcevic@vef.hr

**Keywords:** cafeteria diet, high-fructose diet, NAFLD, fatty acids

## Abstract

The objective of this study was to evaluate the influence of high-fat (HF) and cafeteria diet (CAF) diets and sex on the metabolism of important fatty acids in the liver and perirenal fat tissue. Dietary treatments induced changes in the fatty acid profile in comparison to the untreated group, but the characteristic differences between treated groups were also observable. The HF diet induced an increase in the content of C16:1*n*-7 and C18:1*n*-7 in the liver phospholipids (PL) and triglycerides (TG) and perirenal fat tissue compared to the control and CAF diet. The CAF diet induced a more drastic decrease in both *n*-3 and *n*-6 polyunsaturated fatty acids (PUFA), including depletion of eicosapentaenoic acid (EPA). The CAF diet also increased the content of *n*-6 docosapentaenoic acid (DPA*n*-6) in the liver and decreased it in the perirenal fat. Sex also had a significant influence on the fatty acid profile, but the variables with the highest differences between the CAF and HF treatments were identical in the male and female rats. In this study, we have established that two dietary models of non-alcoholic fatty liver disease (NAFLD) led to characteristic changes in the hepatic and perirenal fat fatty acid profile, in contrast to the control diet and in comparison with each other. These differences could play an important role in the interpretation of the experimental results of nutritional studies.

## 1. Introduction

Non-alcoholic fatty liver disease (NAFLD) is simple to severe steatosis and consequent hepatitis, fibrosis, and cirrhosis [1]. In some cases, NAFLD may progress further to hepatocellular carcinoma [2]. The importance of NAFLD lies in the trend of its increasing prevalence, alongside diabetes and metabolic syndrome [3]. The exact pathology of NAFLD is still unclear, and therefore animal models still play an important role in attempts to elucidate NAFLD pathogenesis and find effective prevention and treatment methods [4]. High-fructose (HF) and high-fat diets (in different forms) are widely used dietary models of rodent NAFLD. One of the possible forms is a “cafeteria diet” (CAF) where animals are allowed free access to highly palatable and energy-dense “unhealthy” human food. The CAF model is a robust rodent model that creates obesity, glucose intolerance, and inflammation [5].

Several polyunsaturated fatty acids (PUFA) have very important and specific biological functions, which makes their abundance in tissue an important health factor [6,7,8,9]. Bioconversion (elongation and desaturation) of these fatty acids is a highly regulated process and includes desaturases and elongases, transcriptional factors, partitioning into oxidation, hormones, metabolites, sex, substrate availability (diet), and microRNAs [10]. Metabolic diseases, such as NAFLD, metabolic syndrome, and diabetes mellitus, affect these regulatory factors, and change the content of tissue fatty acids and their corresponding ratios.

Rodent models are often used without considering the influence of different models on the metabolism of specific fatty acids. Therefore, our aim was to investigate the influence of HF and CAF rodent models on the metabolism of specific fatty acids, which could have important health effects and interfere with the results of an investigation. Additionally, we investigated how initial sex differences in the tissue fatty content, which exist between males and females, are reflected in the fatty acid content during NAFLD progression.

## 2. Materials and Methods

### 2.1. Animals, Feed, and NAFLD Induction

**Figure 1:**
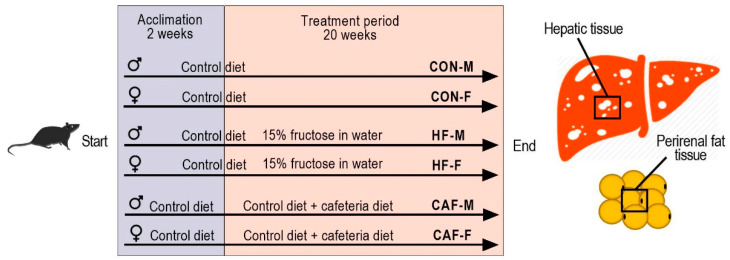
Experimental groups and treatments. CON, control diet; HF, high-fructose diet; CAF, cafeteria diet; M, males; F, females.

Male Wistar HAN rats (18 males 177 ± 6 g and 18 females 140 ± 4 g) were housed, three rats/cage (polycarbonate cage, Techniplast, Buguggiate, Italy), in a 12 h light/dark cycle in the animal laboratory facility of the Institute for Medical Research and Occupational Health, Zagreb, Croatia. The Croatian National Ethics Committee and the Ministry of Agriculture, Republic of Croatia (authorization EP 13/2015), approved all the experimental protocols. After an acclimation period of two weeks, the rats were randomly assigned to one of the experimental diets (Figure 1) to induce NAFLD. The composition of the control diet (standard rat diet: 20% crude protein, 5% crude fat, and 5% crude fiber) and the CAF diet were described previously [5,10]. We followed both diets in detail, with the exception that the meat products were omitted from the CAF diet. The final diet for the CAF groups consisted of the CAF diet (in excess) and the control diet (ad libitum). High-fructose groups (HF) received the control diet and 15% of fructose in their drinking water. Access to food and water (tap water) was unlimited to all rats during the experiment.

### 2.2. Sample Collection and Preparation

All rats were weighed daily at the same time (8 h) using an electronic balance. Simultaneously, fasting blood glucose levels were determined using an Accu-Chek Go [11]. The rats were sacrificed at the 20th week under Narketan/Xylapan anesthesia (Narketan, 80 mg kg^−1^ b.m. and Xylapan, 12 mg kg^−1^ b.m., i.p., Vetoquinol, Bern, Switzerland) followed by exsanguination. Blood was collected by cardiac puncture into ethylenediaminetetraacetic acid (EDTA) tubes, and plasma was obtained by centrifugation at 1500× *g* for 5 min. Immediately after the animals were sacrificed, hepatic and perirenal fat tissues were excised and divided into three parts, which were: (a) stored in 4% neutral buffered formaldehyde for histological assessment, (b) stored in a RNA preserving agent (RNAlater, Thermo Fisher Scientific, Waltham, MA, USA) for extraction of mRNA and (c) frozen at −80 °C for fatty acid analyses.

### 2.3. Serum Biochemistry, Insulin and Insulin Sensitivity

Serum biochemistry parameters (glucose, total cholesterol and triglycerides) were measured by a semi-automatic analyzer (SABA 18, AMS, Rome, Italy). Insulin concentration was determined using a commercial kit (Rat insulin kit ELISA, Mercodia, Upsalla, Sweden). Insulin sensitivity were measured as HOMA-IR [12] and QUICKI [13] indices, using the following equations:

HOMA-IR = (fasting insulin × fasting glucose/2.25(1)

QUICKI = 1/(log(fasting insulin) + log (fasting blood glucose))(2)

### 2.4. Liver Histology

Routine histology was used to produce tissue sections for histological analyses of the rat livers. For semi-quantitative assessment of histopathological alterations to the rat liver, we adopted and extended methods previously described [14].

In our proposed model, a semi-quantitative and quantitative approach was used. Pathological changes were classified into six reaction patterns: steatosis, glycogen storage, atrophy, apoptosis, necrosis, and nuclear alterations. Histopathological alterations of the liver tissue were given one of two assessment values: (1) focal appearance-minimal pathological importance (easily reversible lesions) (2) diffuse appearance-moderate pathological importance (reversible lesions if the stressor is neutralized), and in the case of steatosis, microvesicular (1) and macrovesicular (2). Secondly, in order to obtain a more detailed semi-quantitative analysis, we introduced a second score (s) for each alteration, ranging from 1–10, depending on the degree of histopathological alteration: 1–3 = control, 4–7 = moderate, 8–10 = severe. For example, a particular tissue could show severe diffuse apoptosis and, as such, be assigned as a = 2 and s = 9. Finally, these two values were multiplied to obtain score values (see equation below). The arithmetic mean of these products (mean scored value) represents the degree of damage to a liver tissue after feeding with the experimental diets, following the calculation principle. Nuclear alterations were calculated by assessing ten high-power microscopic fields (×400) in ten random and non-overlapping fields for each specimen.

(3) $$SV\left(liver\right)\;=\;\underset{\left(liver\right)}{\sum }\left(a\left(liver\right)\;\times \;s\left(liver\right)\right)\;$$

### 2.5. Quantitative PCR

The isolation of total RNA from 30 mg of liver tissue was performed with an SV Total RNA Isolation System (Promega GMBH, Mannheim, Germany), according to the manufacturer’s instructions. Total RNA quantity and purity were measured by spectrophotometry using BioDrop (BioDrop μLITE, BioDrop, Cambridge, UK). Reverse transcription and quantification of isolated RNA were performed by One-Step SYBR PrimeScript RT-PCR Kit II (Perfect Real Time, TaKaRa Bio Inc. Shiga, Otsu, Japan) according to the manufacturer’s manual, using a Stratagene MxPro3005 (Agilent Technologies, US and Canada) thermocycler. The oligonucleotides used for quantitative PCR were: CD36-F, 5′-gcctcctttccaccttttgt-3′, CD36-R, 5′-gattcaaacacagcatagatggac-3′, TGF-β1-F, 5′-aatacgtcagacattcgggaagca-3′, TGF-β1-R, 5′- tcaatgtacagctgccgtac-3′, IL6-F, 5′-tgatggatgcttccaaactg-3′, IL6-R, 5′-gagcattggaagttggggta-3′, β-Actin-F, 5′-actattggcaacgagcggtt-3′, β-Actin-R, 5′-tgtcagcaatgcctgggtac-3′, Cyclophilin-F, 5′-cttcttgctggtcttgccattcct-3′, Cyclophilin-R, 5′-ggatggcaagcatgtggtctttg-3′. Gene expression was relatively quantified using the ΔΔCt method (2^ΔΔ^Ct) after it was normalized to the expression level of the housekeeping genes. [15] Data are presented as the fold change in the gene expression relative to the corresponding control group.

### 2.6. Lipid Analyses

Total lipids from the hepatic and perirenal fat tissue were extracted using a chloroform/methanol mixture (2:1, *v*/*v*) [16]. The lipids were dried under N_2_, dissolved in the same mixture (150 µL) with the addition of 0.3 mg/mL BHT, and stored at −80 °C. Total lipids were further separated by solid-phase extraction on a 500 mg aminopropyl bonded silica cartridge (Supelclean, Supelco, Bellefonte, PA, USA) into triglycerides (TG) and phospholipids (PL). A mixture of chloroform/isopropanol (2:1, *v*/*v*) was used for neutral lipids and methanol for phospholipids. After separation, TG and PL were dried, dissolved, and stored as described above.

The fatty acids from the hepatic TG and PL were methylated using 2M KOH in methanol for 15 min at room temperature. The resulting fatty acid methyl esters (FAME) were extracted in n-hexane and transferred into vials for gas chromatography – mass spectrometry (GC-MS) analyses. The FAME was analyzed by GC-MS (QP2010 Ultra, Shimadzu, Kyoto, Japan), using a BPX70 capillary column (0.25 mm internal diameter, 0.25 μm film thickness, 30 m long, SGE, Austin, TX, USA) and adopting previously established conditions. [10] Quantification was performed using methyl tricosanoate (C23:0) and 1,2-dinonadecanoyl-sn-glycero-3-phosphocholine (19:0PC) as internal standards [17]. The fatty acid composition was expressed as the mole percentage of the total fatty acids.

The liver triglyceride content was determined by spectrophotometry, measuring the concentration of glycerol released after lysing the cells and hydrolyzing the triglyceride molecules. The triglyceride concentration was then determined by the glycerol values [18].

### 2.7. Statistical Analyses

The number of animals per group (*n* = 6) was based on power analysis performed with estimates of variation drawn from our previous data, power of 0.8 and *p* ≤ 0.01. The content of the fatty acids and insulin sensitivity indices were considered as the principal variables for power calculations.

All experimental results were analyzed using the GraphPad Prism 8 software v. 8.4.3. (GraphPad Software Inc., San Diego, CA, USA), and data were expressed as the means ± standard deviation (SD). Normality of distribution was tested using the Shapiro-Wilks test. ANOVA and the post-hoc Tukey test were used to determine statistical differences between the group means. Standard deviations of fold changes for the tested gene expression were determined according to the established procedure [15]. Significant differences were considered at *p* < 0.05.

## 3. Results

### 3.1. Insulin Resistance Confirmation

**Figure 2:**
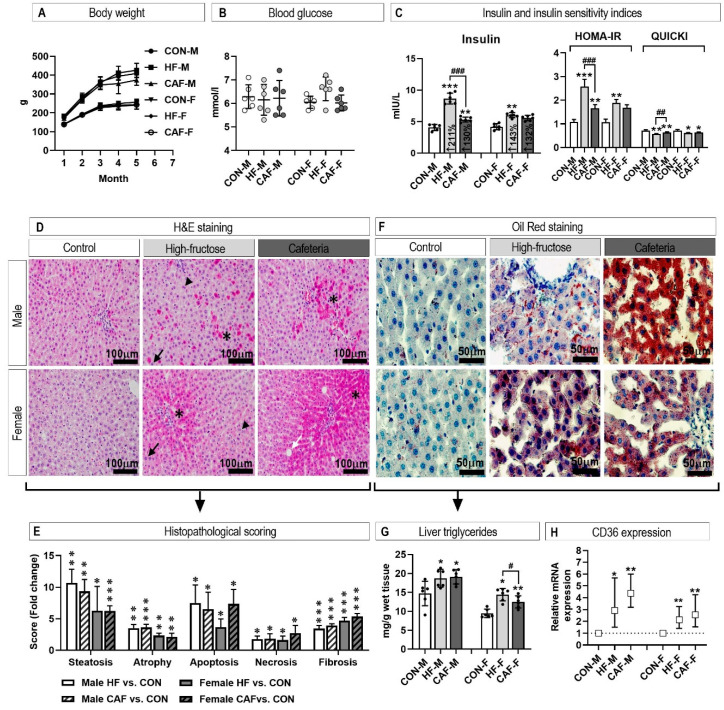
The influence of the treatments on body weight (**A**) and fasting blood glucose values (**B**). Insulin sensitivity was assessed as insulin concentration and insulin sensitivity indices, HOMA-IR and QUICKI (**C**). Photomicrographs of rat livers stained with hematoxylin and eosin (H and E) (**D**) and Oil-red (**F**). In High-fructose treated rats, note areas with apoptosis (asterisk), kariomegaly (arrow), and multinucleated hepatocytes (arrow head), and in Cafeteria diet treated rats, note areas with focal or diffuse apoptosis and necrotic cells (star) and focal macrovesicular steatosis (white arrow). Histopathological scoring is presented as fold change versus the control group (**E**). Lipid accumulation was further assessed by measuring hepatic triglyceride content (**G**) and hepatic CD36 expression. * *p* < 0.05, ** *p* < 0.01, *** *p* < 0.001 for the treatment versus the Control within the same sex. ^#^ *p* < 0.05, ^##^ *p* < 0.01, ^###^ *p* < 0.001 for the HF diet versus the CAF diet within the same sex. *n* = 6 for each group. CON, control diet; HF, high-fructose diet; CAF, cafeteria diet; M, males; F, females.

During the experimental period, there were no differences in body weight and blood glucose between the animals assigned to the different experimental groups (Figure 2A,B). Nevertheless, insulin values increased in the CAF and HF groups. The CAF and HF diets affected insulin sensitivity as measured by HOMA-IR and QUICKI indices (Figure 2C). Insulin sensitivity was more affected in the male HF group compared to the CAF group.

### 3.2. Liver Histology

Hepatic tissue injury was assessed by scoring liver sections stained with hematoxylin and eosin (HE) on a ten-stage scoring system (1–10). After 20 weeks of the experiment, the scores for all investigated abnormalities (steatosis, atrophy, apoptosis, necrosis, and fibrosis) were significantly higher in treated rats compared to the controls (Figure 2D,E). CAF and HF diets induced similar changes, and only the apoptosis score was higher in the CAF diet compared to the HF diets in female rats. The increased lipid accumulation in the treated groups was further demonstrated by Oil red staining (Figure 2F), liver triglyceride content (Figure 2G), and increased CD36 expression (Figure 2H).

### 3.3. Inflammation

**Figure 3:**
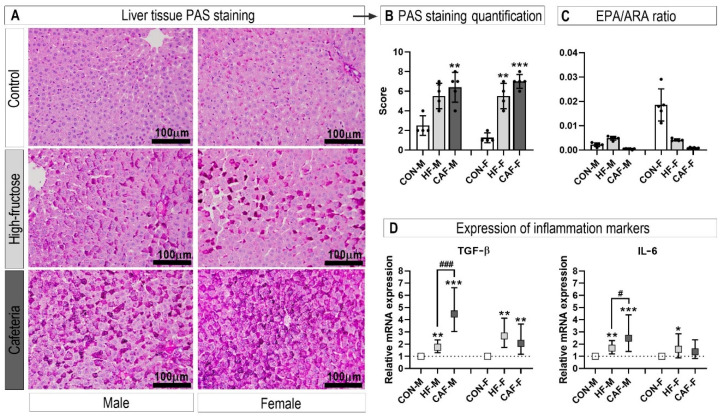
Photomicrographs showing a comparison of the storage products in the livers from the experimental rats (**A**). The appearance of hepatocytes stained with periodic acid-Schiff reagent (PAS), showing glycogen accumulation (glycogen stains magenta and nuclei are blue) and quantification of glycogen accumulation (**B**). Eicosaenoic/arachidonic (EPA/ARA) ratio suggesting a shift to more pro-inflammatory eicosanoids (**C**). The gene expression of the inflammation markers (Transforming growth factor-β and interleukin-6, TGF-β and IL-6) was assessed by the quantitative PCR (**D**). * *p* < 0.05, ** *p* < 0.01, and *** *p* < 0.001 for the treatment versus the Control within the same sex. ^#^ *p* < 0.05, and ^###^ *p* < 0.001 for the HF diet versus the CAF diet within the same sex. *n* = 6 for each group. CON, control diet; HF, high-fructose diet; CAF, cafeteria diet; M, males; F, females.

We next investigated the inflammatory status of the hepatic tissue since low-level chronic inflammation is characteristic of animal obesity models. The hepatocytes of the treated rats, stained with PAS staining, showed increased glycogen accumulation consistent with an inflammatory process (Figure 3A,B). The eicosapentaenoic acid (EPA, less inflammatory products)/arachidonic acid (ARA, more inflammatory products) ratio was significantly decreased in male and female rats on the CAF diets compared to the control and the HF diets (Figure 3C). The expression of the inflammation markers additionally confirmed the inflammatory state (Figure 3D). An interesting observation was the further increase in the expression of inflammation markers in the CAF diet in contrast to the HF diet in the male rats.

### 3.4. Hepatic and Perirenal Fat Fatty Acid Profile

**Figure 4:**
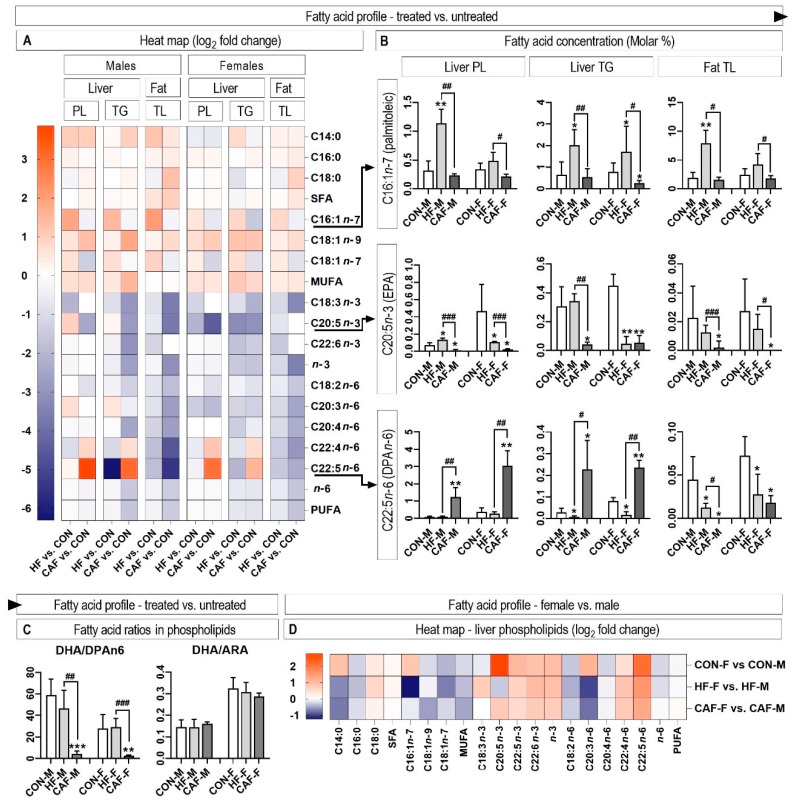
The influence of the treatments on the fatty acid profile of the hepatic phospholipids and triglycerides and perirenal total fat is presented as a heat map (**A**). The most characteristic fatty acids (C16:1*n*-7, C20:5*n*-3 and C22:5*n*-6) are presented as the molar percentage (**B**). Additionally, two important ratios of DHA/DPA*n*-6 and DHA/ARA are presented (**C**). The influence of sex in different treatments is presented as a heat map (**D**). * *p* < 0.05, ** *p* < 0.01, and *** *p* < 0.001 for the treatment versus the Control within the same sex. ^#^ *p* < 0.05, ^##^ *p* < 0.01, and ^###^ *p* < 0.001 for the HF diet versus the CAF diet within the same sex. *n* = 6 for each group. PL, phospholipids; TG, triglycerides; TL, total lipids. CON, control diet; HF, high-fructose diet; CAF, cafeteria diet; M, males; F, females.

The fatty acid profile of hepatic and perirenal fat tissue is presented in a heat map, which clearly shows the influence of the treatment (Figure 4A). In general, it could be observed that there was a significant difference between the CAF diet and the HF diet. The two most important differences were that the HF diet induced an increase in the content of monounsaturated fatty acids (MUFA), while the CAF diet induced a strong decrease in both *n*-3 and *n*-6 polyunsaturated fatty acids (PUFA). We further showed the concentrations of three fatty acids that were the most characteristic in distinguishing the experimental groups (Figure 4B). The content of palmitoleic fatty acid (C16:1*n*-7) only increased in the HF groups compared to the control in the liver PL and TG and perirenal fat tissue. The content of eicosapentaenoic acid (EPA) was almost depleted in the CAF groups in all tissues examined. Finally, the content of n6 docosapentaenoic acid (DPA*n*-6) increased strongly in the CAF treated rats in the hepatic tissue, while its content decreased in the perirenal fat tissue. In the CAF treated rats, the large increase in DPA*n*-6 content, alongside a moderate decrease in docosahexaenoic acid content, resulted in the drastic decrease in the DHA/DPA*n*-6 ratio in the hepatic phospholipids (Figure 4C). Meanwhile, the docosahexaenoic/arachidonic (DHA/ARA) ratio remained constant in all the examined rats.

**Figure 5:**
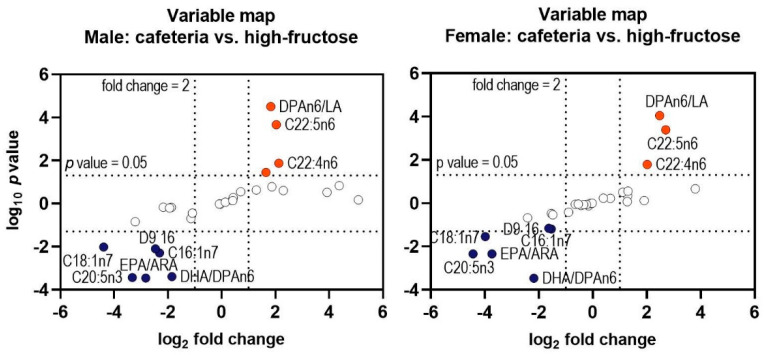
Variable map showing the variables with the highest significance between the Cafeteria diet and High-fructose diet in the male and female rats. Down-regulation << Average ratio >> Up-regulation. LA, linoleic acid.

Sex had a significant influence on the fatty acid profile of the liver phospholipids (Figure 4D). Female rats had higher concentrations of *n*-3 fatty acids and *n*-6 fatty acids with 22 carbon atoms, while the concentration of *n*-6 fatty acids with 18 and 20 C atoms was lower in the female rats. Interestingly, the contents of C16:1*n*-7 and C20:3*n*-6 showed a treatment × sex interaction (Figure 4D). Nevertheless, besides the initial sex differences, the variables with the highest differences between the CAF and HF treatments were identical in the male and female rats (Figure 5).

## 4. Discussion

Diet-induced obesity is widely used to examine metabolic syndrome and its hepatic manifestation, NAFLD, in rodent translational models [4]. Both models included in our trial showed changes characteristic for NAFLD. The fasting glucose was not significantly increased by the treatments, but treated animals showed increased fasting insulin concentrations. Moreover, insulin sensitivity, measured as HOMA-IR and QUICKI indices, was affected by the treatments in both sexes. HOMA-IR and QUICKI cannot be considered as a replacement for direct measurement of insulin resistance, but they provide reliable approximation in rodent models and humans [19]. Interestingly, insulin content and insulin sensitivity indices were more affected by the HF diet than the CAF diet in the male rats. The pathohistological analysis further confirmed NAFLD because the CAF and HF treated rats developed the characteristic pathological abnormalities in the hepatic tissue. Both treatments and sexes developed similar levels of pathohistological abnormalities in the hepatic tissue, including an increased level of steatosis, fibrosis, atrophy, apoptosis, and necrosis. The pathological changes in the livers of NAFLD rodent models are characteristic, but the severity of changes varies according to the trial duration, and dietary intervention used [20,21]. The third feature of NAFLD examined was chronic low-grade inflammation, which is now considered one of the key features of metabolic syndrome. The histological measurement of inflammation (glycogen accumulation measured as PAS staining) was further confirmed by quantitative PCR analyses of the pro-inflammatory genes, IL-6 and TGF-β. Both measured genes had higher expression in male rats fed the CAF diet compared to the HF diet, suggesting a higher degree of inflammation processes. In an effort to explain these results, two important variables in experimental NAFLD should be mentioned. When comparing the effects of a high-fat diet, a high-fructose diet, and high-fructose/high-fat/high-cholesterol diets on the development of NAFLD in rats, it may be found that diets with a higher fat content are more effective in induction of NAFLD [21]. Secondly, most experimental NAFLD studies using rodent models with dietary interventions have found that NAFLD is more severe in male animals [22].

To investigate whether these variables also influence fatty acid metabolism, we performed comprehensive analyses of fatty acids in the hepatic and perirenal fat tissue. These analyses showed the strong influence of both dietary treatments on the quantity and ratios of different fatty acids in comparison to the untreated groups. Nevertheless, significant differences also existed between the treated groups (high-fructose vs. cafeteria diet). The usual rodent Control diets comprise high amounts of carbohydrates (starch and dextrin). Therefore, some authors consider these control diets as high carbohydrate diets [23]. Other authors consider feed or water supplemented with various amounts of sugars (fructose, glucose, or sucrose) as a high-carbohydrate diet [20,24]. Consequently, both diets are known as “high carbohydrate”, but they differ greatly in the type of carbohydrate. In some cases, experimental diets are not isonitrogenous, which further complicates the comparison of results from different studies [23]. Moreover, sometimes the exact carbohydrate content and type are not presented, and, therefore, it could only be assumed that the high-carbohydrate diet was created by replacing starch in the control diet with another type of carbohydrate [25]. The problem is outlined in experiments where several differences, including weight gain and insulin resistance, exist between the control groups (standard chow vs. low-fat diet) [5]. The authors observed that the effect of different control feeds could vary widely and emphasize the importance of feed composition and ingredients in obesity studies.

All experimental groups in our trial, untreated, HF treated, and CAF treated, had the characteristic patterns of changes in the fatty acid profiles, with characteristically distinguishing fatty acids. MUFA content was characterized by a significant increase in the content of palmitoleic acid in only the HF treated group. Moreover, for the same acid, we found a significant treatment × sex interaction. Palmitoleic acid, which is nowadays considered to be lipokine [9], normally has low concentrations in tissues due to β-oxidation [26], but it responds to increased Δ9-desaturation during a high carbohydrate diet [20]. In that sense, an increase in palmitoleic acid in the HF treated group could be expected. In the CAF treated groups, *de novo* lipogenesis did not increase due to the high amount of dietary fat, and consequently, the content of palmitoleic acid did not increase. The previously observed correlation between the serum C16:1*n*-7/C16:0 ratio and liver inflammation, steatohepatosis and fibrosis, suggests that this ratio could be an appropriate method for non-invasive diagnosis of non-alcoholic steatohepatitis [27]. In rodent models, due to the influence of diet, the C16:1*n*-7/C16:0 ratio is applicable in rats treated with the HF diet, but not in the CAF treated rats. Therefore, it would be advisable to test if the applicability of the C16:1*n*-7/C16:0 ratio is also diet-dependent in humans.

Concerning the PUFA content, it should be noted that most investigations that used a high content of carbohydrate and/or fat to induce NAFLD (metabolic syndrome, obesity) are consistent in observation of a decrease in essential fatty acids, linoleic and linolenic [20,24,28,29,30,31]. Nevertheless, there is much less consistency in the quantity of fatty acids with more C atoms and/or double bonds. These apparent differences are the consequence of the interaction between the complex regulation of fatty acid bioconversion and the different dietary factors present in NAFLD/obesity/metabolic syndrome trials.

In our trial, the characteristic PUFA was EPA, which, together with the DHA, represents an important *n*-3 PUFA with extensive health effects [32]. The CAF treatment almost completely depleted the hepatic tissue of EPA, which consequently decreased the EPA/ARA ratio. Due to the differences in the eicosanoids products of EPA and ARA, the decreased EPA/ARA ratio could suggest a shift to the production of more pro-inflammatory eicosanoids and increased susceptibility to disease complications [33,34].

Another characteristic PUFA for CAF treatments was DPA*n*-6, whose content increased greatly and caused a decrease in the phospholipid DHA/DPA*n*-6 ratio. When the DHA concentration decreases, a parallel increase in DPA*n*-6 is evident, which makes the DHA/DPAn6 ratio a possible index of DHA deficiency [35]. It is interesting that in our trial DPA*n*-6 increased with a simultaneous very low decrease in DHA content. Nevertheless, it could be reasonably assumed that after a long experimental period and/or a larger amount of fructose, a further decrease in DHA content may be expected. In contrast to the hepatic phospholipids, DPA*n*-6 concentrations decreased in the perirenal fat tissue suggesting that, at least to some extent, redistribution of DPA*n*-6 among the lipid deposits could exist.

## 5. Conclusions

In this study, we established that the two dietary models of NAFLD develop characteristic changes in the hepatic and perirenal fat fatty acid profile, in contrast to the control diet and between each other. The most characteristic differences between these models include the content of EPA, DPA*n*-6, palmitoleic and cis-vaccenic acid, and the related ratios. In addition, it was observed that the Cafeteria diet led to more extensive depletion of the PUFA. Sex had a significant influence on hepatic and perirenal fat fatty acid profiles, but the most significant differences between the High-fructose diet and Cafeteria diet fed rats were identical in male and female rats. These studies support the observation that the differences in the fatty acid profile in response to the diet used to induce NAFLD could play an important role in the interpretation of the experimental results.
